# Obesity, diabetes and the risk of colorectal adenoma and cancer

**DOI:** 10.1186/s12902-019-0444-6

**Published:** 2019-10-29

**Authors:** Ghodratollah Soltani, Arash Poursheikhani, Maryam Yassi, Abdorasool Hayatbakhsh, Matin Kerachian, Mohammad Amin Kerachian

**Affiliations:** 1 Cancer Genetics Research Unit, Reza Radiotherapy and Oncology Center, Mashhad, Iran; 2 Medical Genetics Research Center, Mashhad University of Medical Sciences, Mashhad, Iran; 3 Department of Medical Genetics, Faculty of Medicine, Mashhad University of Medical Sciences, Mashhad, Iran; 4 Faculty of Medicine, McGill University, Montreal, Canada; 5 Research Institute at McGill University Health Center, Montreal, Canada

**Keywords:** Cancer, Obesity, Type 2 diabetes, Overweight, Epidemiology, Diabetes

## Abstract

**Background:**

Colorectal cancer (CRC) is the fourth most commonly diagnosed gastrointestinal (GI) malignancy and the third leading cause of cancer-related death worldwide. In the current case-control study, an association between diagnosis of CRC, obesity and diabetes was investigated.

**Methods:**

Demographic characteristics, colonoscopy reports, history of drug, smoking, and medical history were collected from patients referred to a colonoscopy unit. The location, size and number of the polyps were recorded during the colonoscopy. Statistically, *t*-test was conducted for mean comparison for the groups. Pearson’s chi-squared test (χ2) was applied to categorize variables. Five classification methods based on the important clinicopathological characteristics such as age, BMI, diabetes, family history of colon cancer was performed to predict the results of colonoscopy.

**Results:**

Overall, 693 patients participated in this study. In the present study, 115 and 515 patients were evaluated for adenoma/adenocarcinoma and normal colonoscopy, respectively. The mean age of patients positive for adenoma or adenocarcinoma were significantly higher than the negative groups (*p* value < 0.001). Incidence of overweight and/or obesity (BMI > 25 kg/m2) were significantly higher in adenoma positive patients as compared to controls (49.9 and 0.9% respectively, *p* value = 0.04). The results also demonstrated a significant association between suffering from diabetes and having colon adenoma (OR = 1.831, 95%CI = 1.058–3.169, p value = 0.023). The experimental results of 5 classification methods on higher risk factors between colon adenoma and normal colonoscopy data were more than 82% and less than 0.42 for the percentage of classification accuracy and root mean squared error, respectively.

**Conclusions:**

In the current study, the occurrence of obesity measured based on BMI and diabetes in the adenoma positive patient group was significantly higher than the control group although there was no notable association between obesity, diabetes and adenocarcinoma.

## Background

Colorectal cancer (CRC) is the fourth most commonly diagnosed gastrointestinal (GI) malignancy and the third leading cause of cancer-related death worldwide [1–3]. It occurs in 5% of the general population at any given time [1]. According to GLOBOCAN 2018, it is the third common cancer in Iran with 9864 new cases in 2018 [4]. The mean direct medical cost of CRC per patient in Iran is more than 16,000 US dollars [5] and thus, it is estimated that its economic burden will range from 175,000,000 to 250,000,000 US dollars in 2019.

The incidence rate of CRC has increased in both developing and Western countries over the last decades [6–8]. The global burden of CRC is expected to increase by 60% to more than 2.2 million new cases and 1.1 million deaths by 2030 [9]. Each year, over 132.000 new cases of CRC are diagnosed in the United States, and approximately fifty thousand patients will pass away from this cancer [10]. The five-year survival rate is above 90% for first stage of the disease [11]. A large number of evidence has revealed that environmental and modifiable factors such as smoking, alcohol, obesity, unhealthful dietary habits, diabetes and physical inactivity have a major impact on the development of CRC [2, 6, 8, 12, 13]. Based on the diabetes country profiles of the World Health Organization (WHO) in 2016, the prevalence rates of physical inactivity, overweight, and obesity in Iran were 31.9, 60.5, and 24.9%, respectively [14]. Generally, unhealthy lifestyles might accounts for up to 70% of CRC etiology [15, 16]. It has been reported that obesity, particularly central obesity is one of the most significant predisposing factors for numerous cancers and chronic diseases [3]. Moreover, it has been shown that obesity is a meaningful contributor to CRC and is considered as a poor prognosis factor in cancer development [11, 17, 18]. On the other hand, losing weight might have desirable effects on the prognosis of the disease [19]. According to the WHO, obesity is defined as a body mass index (BMI): normal weight (BMI: 18.5–24.9 kg/m^2^), overweight (BMI: 25.0–29.9 kg/m^2^), obesity (BMI: 30.0–34.9 kg/m^2^), severe obesity (BMI: 35.0–39.9 kg/m^2^) and for morbid obesity (BMI ≥40 kg/m^2^) [1, 2, 20]. Approximately 30% of the American population is classified in the overweight or obese category [21]. Obesity could be associated with obesity-related cancers such as breast, liver, gynaecological, oesophagus, kidney, lung, pancreatic, thyroid, gallbladder and CRC [6, 21]. It initiates different cellular and molecular pathways, which eventually lead to tumor formation. Adipose tissue produces many kinds of hormones and pro-inflammatory cytokines, among them, interleukin 6, tumor necrosing factor-α, leptin and adiponectin provide desirable inflammatory microenvironment conditions for cancerous cells [22, 23]. Current studies have revealed that adipose tissue stimulates proliferation, migration, angiogenesis and oxidative stress induction [21]. In a recent meta-analysis by Dong et al., it was demonstrated that abdominal obesity is highly associated with an increased relative risk of CRC [6].

In addition to obesity, insulin resistance and hyper-insulinaemia, are also associated with CRC [17, 24]. Insulin resistance and insulin response are highly correlated since majority of the insulin-resistant individuals are either in the highest insulin response quartile or the second highest [25]. Besides, numerous epidemiological studies depict that CRC is more prevalent among diabetic patients as compared to non-diabetic ones [26]. Several observations have elucidated that there is an association between diabetes and an elevated incidence ratio of cancer in specific organs such as liver, pancreas, endometrial, breast, bladder, and colon. Aberration in insulin regulation underlies both diabetes and obesity-related tumorigenesis through several signalling pathways such as insulin-like growth factor (IGF)-1 receptors [27, 28].

In the current study, we investigated an association between diagnosis of CRC, obesity and diabetes in the selected group of CRC patients.

## Methods

The study population was collected from patients referred to the colonoscopy unit of Reza Radiotherapy and Oncology Centre, Mashhad, Iran from May 2015 to October 2017. Patients with symptoms of colon cancer include changes in bowel movements, rectal bleeding, anemia, losing weight when not in diet, loss of appetite, nausea or vomiting, persistent abdominal discomfort such as cramps, gas or pain.

Patient samples in case group (*N* = 178) had a diagnosis determined by colonoscopy and confirmed by pathology. Control group (*N* = 515) were taken from individuals who underwent CRC screening by colonoscopy that was negative for polyps and CRC through the entire colon and rectum.

All the subjects filled out the administered questionnaires before their colonoscopy. The present study was approved by Mashhad University of Medical Sciences (MUMS) ethic committee (approval #940358) confirming that authors obtained consent to publish from the participants. All methods were performed in accordance with the relevant guidelines and regulations. Excluding criteria of the study were patients with previous CRC, positive familial history of adenoma polyposis, inflammatory bowel disease, hereditary CRC and patients with incomplete colonoscopy and documentations. Demographic characteristics, colonoscopy reports, history of drug (opium) and smoking, as well as medical history were all collected. Weight and height were measured and BMI was calculated and patients were subsequently classified according to WHO benchmarks.

Besides, the location, size and number of the polyps were recorded during the colonoscopy. The polyps were classified as conventional adenomas and serrated lesions. The location of lesion was defined as anal, rectum, sigmoid, transverse colon, descending colon, ascending colon, and cecum. Based on histological classification, the two major classes of colorectal polyps were conventional adenomas including tubular, tubulovillous or villous adenomas and serrated lesions including hyperplastic, sessile serrated polyps or traditional serrated adenomas [29]. Histopathological characteristics of polyps were determined by two expert gastroenterology pathologists. Individuals who had a colonoscopy for their first time with no symptoms were considered as “screening colonoscopy participants”. Patients who had previously colonoscopies with polyps removed and admitted for follow-up were so-called “follow-up colonoscopies”. Patients undergoing colonoscopies for symptoms such as abdominal pain or rectal bleeding were defined as “diagnostic colonoscopies” [30].

### Statistical analysis

The data were presented as mean and standard deviation (SD). The *t*-test was conducted for all the variables, which had a parametric distribution in order to compare means in case and control groups. Pearson’s chi-squared test (χ2) was applied to categorize variables. As a result, risk factors were defined between normal colonoscopy and case groups with *p*-values < 0.05 which was considered statistically significant. The classification process was performed by using multiple classification models that were designed to classify the normal colonoscopy and adenoma positive cases based on important attributes between normal colonoscopy and adenoma positive. The classification methods consisted of decision tree [DT: Decision tree classifier is a rule-based classifier that is the most powerful and popular tool for classification and prediction. A Decision tree is a flowchart like tree structure, where each internal node denotes a test on an attribute, each branch represents an outcome of the test, and each leaf node (terminal node) holds a class label], random forest [RF: Random forests are made of many decision trees. They are ensembles of decision trees, each decision tree created by using a subset of the attributes used to classify a given population. Those decision trees vote on how to classify a given instance of input data, and the random forest bootstraps those votes to choose the best prediction. This is done to prevent overfitting, a common flaw of decision trees], neural network [NN: Neural networks are based on the operation and structure of the human brain. These networks process one record at a time and “learn” by comparing their classification of the record (which as the beginning, is largely arbitrary) with the known actual classification of the record. Neural networks are typically organized in layers. Layers are made up of a number of interconnected ‘nodes’, which contain an ‘activation function’. Patterns are presented to the network via the ‘input layer’, which communicates to one or more ‘hidden layers’ where the actual processing is done via a system of weighted ‘connections’], K-nearest neighbour [Knn: K-nearest neighbour is one of the most popular and most important algorithms. KNN is known to be very simple and easy. KNN is an example-based learning group. This algorithm is also one of the lazy learning techniques. KNN is done by searching for the group of K objects in the closest training data (similar) to objects in new data or data testing. Generally, the Euclidean distance formula is used to define the distance between two training objects and testing] and Support vector machine [SVM: Support vector machine is another popular classification method. Initially SVM map the input vector into a feature space of higher dimensionality and identify the hyperplane that separates the data points into two classes. The marginal distance between the decision hyperplane and the instances that are closest to boundary is maximized. The resulting classifier achieves considerable generalizability and can therefore, be used for the reliable classification of new samples. It is worth noting that probabilistic outputs can also be obtained for SVM. The identified hyperplane can be thought as a decision boundary between the two clusters. Obviously, the existence of a decision boundary allows for the detection of any misclassification produced by the method] [31, 32].

In a nutshell, by using these classification methods based on the important features including age, BMI, diabetes, family history of colon cancer, drug abuse, we can predict the result of colonoscopy (normal colonoscopy- adenoma positive). All the cases were matched to control with regards to age and weight. In this study, the statistical analysis results were analysed with R programming and Waikato Environment for Knowledge Analysis (Weka) Toolkit.

## Results

**Fig. 1:**
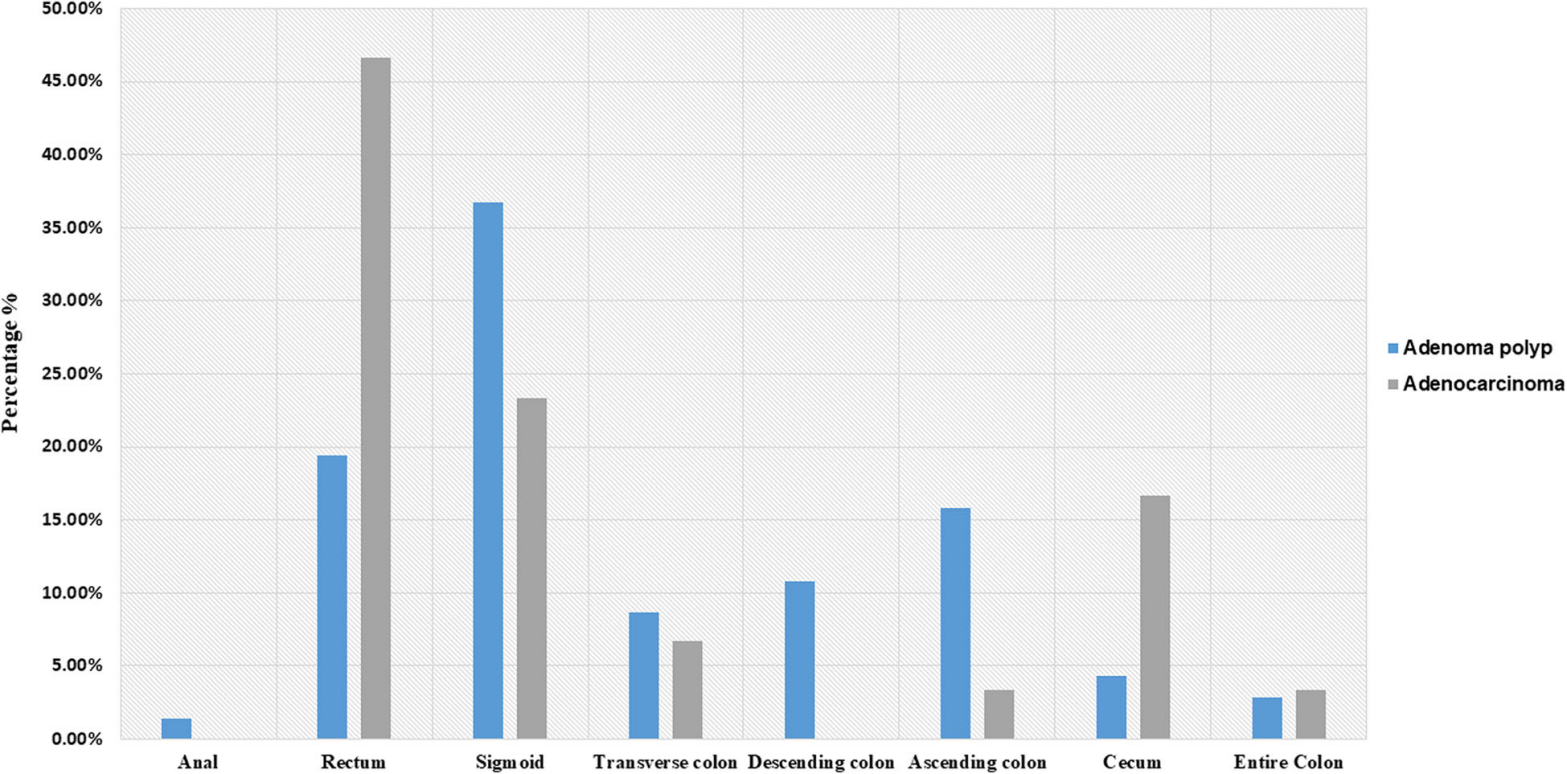
Location of Polyp/ Tumor in colon. Most patients had polyps or tumors in the rectosigmoid although it was not statistically significant in compare to other locations (*p* value = 0.1615)

Overall, 693 patients were participated in this study with mean (SD) age of 49.84 (14.63). Almost half of the patients 347 (51%) were females. Approximately 446 (65%) of participants had BMI > 25 kg/m^2^ and 553 (79.9%) of patients had a positive medical history (Table 1). About 570 (82.2%) of patients had a family history of different cancers. Nearly 90 % of patients were regular smokers and 30 (3.1%) and 53 (7.6%) of subjects had a positive history of alcohol and drug abuse, respectively. The colonoscopy indications were screening 36 (5.2%), follow-up 116 (16.7%) and diagnostic 541(78.1%). The pathological interpretation of colonoscopy biopsies were normal in 515 (74.3%) of cases, tubular adenoma in 92 (13.3%), adenocarcinoma in 30 (4.3%), tubulovillous adenoma in 21 (3%), hyperplastic polyp in 14 (2%), benign polyp in 13 (1.9%), sessile serrated adenoma in 4 (0.6%), villous adenoma in 2 (0.3%) and traditional serrated adenoma in 2 (0.3%) of the patients. Altogether, positive polyps and positive adenomas composed 149 (21.5%) and 115 (16.6%) of the patients, respectively. The adenomas were mostly located in sigmoid 51 (36.7%) and rectum 27 (19.4%) (i.e. left-sided of GI tract) of the patients. Furthermore, Most patients (58.57%) had polyp or tumor in rectum and sigmoid (rectosigmoid) and only 41.42% of cases had polyp or tumor in the rest of colon which was not statistically significant (*p* value = 0.1615) (Fig. 1). Adenoma with the size ≥1 cm was observed in 101 (87.8%) of participants. Patient and histopathological characteristics were presented in Table 1.

**Table 1:** Clinicopathological feature of patient

Variable	Number	Results, n (%)
Age (years), mean (SD)	693	49.84 (14.63)
Gender		
Male	693	346 (49)
Female		347 (51)
Indication		
Screening	693	36 (5.2)
Follow-up		116 (16.7)
Diagnostic		541 (78.1)
BMI (kg/m^2^)		
< 18.5	693	34 (4.9)
18.5–24.9		211 (30.4)
25.0–29.9		260 (37.5)
30.0–34.9		143 (20.6)
= > 35.0		45 (6.5)
Medical history of any disease		
Anemia		102 (14.7)
Asthma		20 (2.9)
Arthritis		19 (2.74)
Thyroid Disorder		43 (6.2)
Type 1 or 2 Diabetes		91 (13.1)
High Blood Pressure		152 (21.9)
High Cholesterol		68 (12.7)
Gastrointestinal symptom		277 (32.76)
Migraine		14 (2)
Heart Attack		79 (11.4)
Kidney Disease		34 (4.9)
Liver Disease		75 (10.8)
Neurological Disease		57 (8.2)
Stomach Colon Disease		68 (9.8)
Nothing		138 (19.9)
Fx of cancer		570 (82.2)
Fx of colon cancer		125 (18.03)
Fx of colon cancer (Relative)		
Mother	125	30 (20.4)
Father		27 (18.4)
Brother		22 (15)
Sister		21 (14.3)
Children		0
Second relative		47 (32)
Personal history of any cancer	693	123 (17.7)
Positive Hx of Drug		53 (7.6)
Positive Hx of Smoking		131 (18.9)
Positive Hx of Alcohol		30 (3.1)
Result of pathology/colonoscopy		
Normal Colonoscopy	693	515 (74.3)
Tubular adenoma		92 (13.3)
Tubulovillous adenomas		21 (3)
Villous adenoma		2 (0.3)
Sessile serrated adenoma /polyp		4 (0.6)
Serrated polyposis = Hyperplastic polyp syndrome		0
Traditional serrated adenoma		2 (0.3)
Hyperplastic polyp		14 (2)
Adenocarcinoma		30 (4.3)
Benign		13 (1.9)
Positive polyp		149 (21.5)
Positive adenoma		115 (16.6)
Adenoma location		
Anal	115	2 (1.4)
Rectum		27 (19.4)
Sigmoid		51 (36.7)
Transvers colon		12 (8.6)
Descending colon		15 (10.8)
Ascending colon		22 (15.8)
Cecum		6 (4.3)
All of the colon		4 (2.8)
Adenoma size ≥1	115	101 (87.8)
High grade dysplasia	115	11 (9.6)

*SD* Standard deviation, *n* Number, *BMI* Body mass index, *ADHD* Attention deficit hyperactivity disorder, *Fx* Family History, *Hx* History

There were 515 participants with normal colonoscopy as compared to 115 adenoma positive patients. The mean age of the two groups was significantly higher in adenoma positive group (*p* value < 0.001). Gender distribution of the groups were not significantly different. Incidence of overweight and/or obesity (BMI > 25 kg/m^2^) were significantly higher in adenoma positive patients as compared to normal ones (49.9 and 0.9% respectively, *p* value = 0.04, Table 2). Interestingly, the incidence of positive history of type 1 or type 2 diabetes observed in the adenoma positive group was significantly higher than the control group (19.1 and 10.9%, respectively with *p* value =0.02). Similarly, the incidence of a positive family history of CRC was dramatically higher in adenoma positive patients compared with normal colonoscopy cases (25.2 and 16.5%, respectively with p value = 0.03). Table 2 describes the association between age, BMI > 25 kg/m^2^, history of diabetes and family history of CRC with the risk of colon adenoma that Odd ratio for overweight/obesity and family history diseases related to CRC was calculated as 1.86; 95%CI, 1.24–2.82 and 1.76; 95%CI, 1.09–2.83, respectively.

**Table 2:** Association of potential risk factors between normal colonoscopy and adenoma positive

	Normal Colonoscopy		Adenoma Positive		p value
	Number	Results	Number	Results	
Age (years), Mean (SD)*	515	47.5 (14.4)	115	55.9 (13.3)	1.48E-08
Male, n (%)		251 (48.7)		65 (56.5)	0.1596
BMI (kg/m^2^), Mean (SD)		27.6 (14.4)		26.4 (5.4)	0.1528
BMI (kg/m^2^) ≥ 25, n (%)*		257 (49.9)		70 (60.9)	0.0429
Positive Hx of type.1.or.2.Diabetes*		56 (10.9)		22 (19.1)	0.023
Positive Hx of colon disease, n (%)		48 (9.3)		12 (10.4)	0.8474
Positive Hx of colon polyp, n (%)		41 (8)		11 (9.6)	0.7056
Positive Hx of colon cancer family*, n (%)		85 (16.5)		29 (25.2)	0.0394
Positive Hx of Drug*, n (%)		23 (4.5)		11 (9.5)	0.05
Positive Hx of Smoking, n (%)		99 (19.22)		21 (18.26)	0.9153

*SD* Standard deviation, *n* Number, *BMI* Body mass index, *Hx* History

*p value< 0.05

In the current study, there were 30 patients with adenocarcinoma. Mean age of patients with adenocarcinoma was higher in comparison with normal group (59.2 for adenocarcinoma and 47.5 for normal colonoscopy, *p* < 0.001). Gender distribution was similar in both groups. Interestingly, the mean BMI of the cancer patient group was lower than the normal group (24.7 kg/m^2^ for adenocarcinoma and 27.6 kg/m^2^ for normal group, *p* = 0.01). Positive history of type 1 or 2 diabetes and colon cancer was not significantly different in these groups as reported in Table 3. By comparing positive lesions (positive adenoma and adenocarcinoma) with the control group for BMI > 25 kg/m^2^ and diabetes *p* values were 0.5 and 0.02, respectively.

**Table 3:** Association of potential risk factors between normal colonoscopy and adenocarcinoma

	Normal Colonoscopy		Adenocarcinoma		*p* value
	Number	Results	Number	Results	
Age (year), Mean (SD)*	515	47.5 (14.4)	29	59.2 (15)	2.2e-16
Male, n (%)		251 (48.7)		18 (62.1)	0.2277
BMI (kg/m2), Mean (SD)*		27.6 (14.4)		24.7 (5.2)	0.01467
BMI (kg/m2) ≥25, n (%)		257 (49.9)		15 (51.7)	0.8486
Positive Hx of type.1.or.2.Diabetes		56 (10.9)		3 (10.3)	0.9290
Positive Hx of colon disease, n (%)		48 (9.3)		4 (13.8)	0.6835
Positive Hx of colon polyp, n (%)		41 (8)		5 (17.2)	0.1601
Positive Hx of colon cancer family, n (%)		85 (16.5)		4 (13.8)	0.8393
Positive Hx of Drug, n (%)		23 (4.5)		3 (10.3)	0.3190
Positive Hx of Smoking, n (%)		99 (19.22)		7 (24.13)	0.6824

*SD* Standard deviation, *BMI* Body mass index, *Hx* History, *n* Number

*p value< 0.05

In this study, several direct and indirect diseases were considered as risk factors of CRC. Direct diseases included anaemia, blood clotting, thyroid disorders, sexually transmitted disorders, type 1 or 2 diabetes, gynaecological diseases, acromegaly, stomach and colon diseases that have direct effect on colon cancer. In contrast, indirect diseases included high blood pressure, high cholesterol, heart and liver diseases that have indirect effect on CRC [11]. The emerged data demonstrated significant association between type 1 or 2 diabetes with the incidence of colon adenoma (DOR = 1.831, 95%CI = 1.058–3.169 *p* = 0.023) (Table 4).

**Table 4:** Direct and indirect disease effect on colon adenoma polyp

	DOR	[95% CI]	*p* value
Direct disease			
Anemia	0.575	0.296–1.119	0.8
Blood Clots	0.889	0.042–18.646	0.4
Thyroid Disorder	0.623	0.239–1.628	0.7
Sexually Transmitted	0.294	0.017–5.176	0.5
Type 1 or 2 Diabetes *	1.831	1.058–3.169	0.023
Gynecological Disease	0.637	0.078–5.225	0.7
Acromegaly	0.492	0.026–9.203	0.8
Gastrointestinal symptom	0.680	0.432–1.071	0.2
Stomach Colon Disease	1.133	0.581–2.210	0.8
Indirect disease			
High Blood Pressure	1.542	0.972–2.446	0.1
High Cholesterol	1.381	0.781–2.441	0.3
Heart Disease	1.325	0.730–2.405	0.4
Liver Disease	1.503	0.822–2.748	0.2

* *p* value< 0.05

**Fig. 2:**
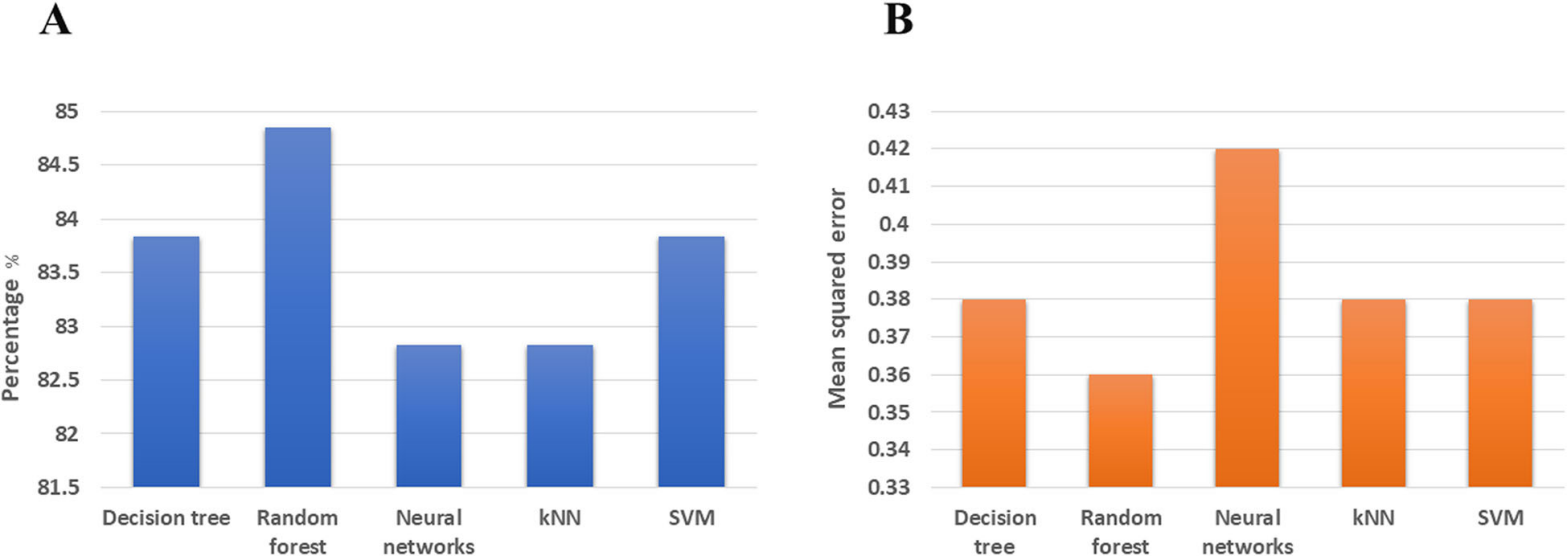
Performance of five classifications. It indicates the classification accuracy (**a**) and the mean squared error (**b**)

**Fig. 3:**
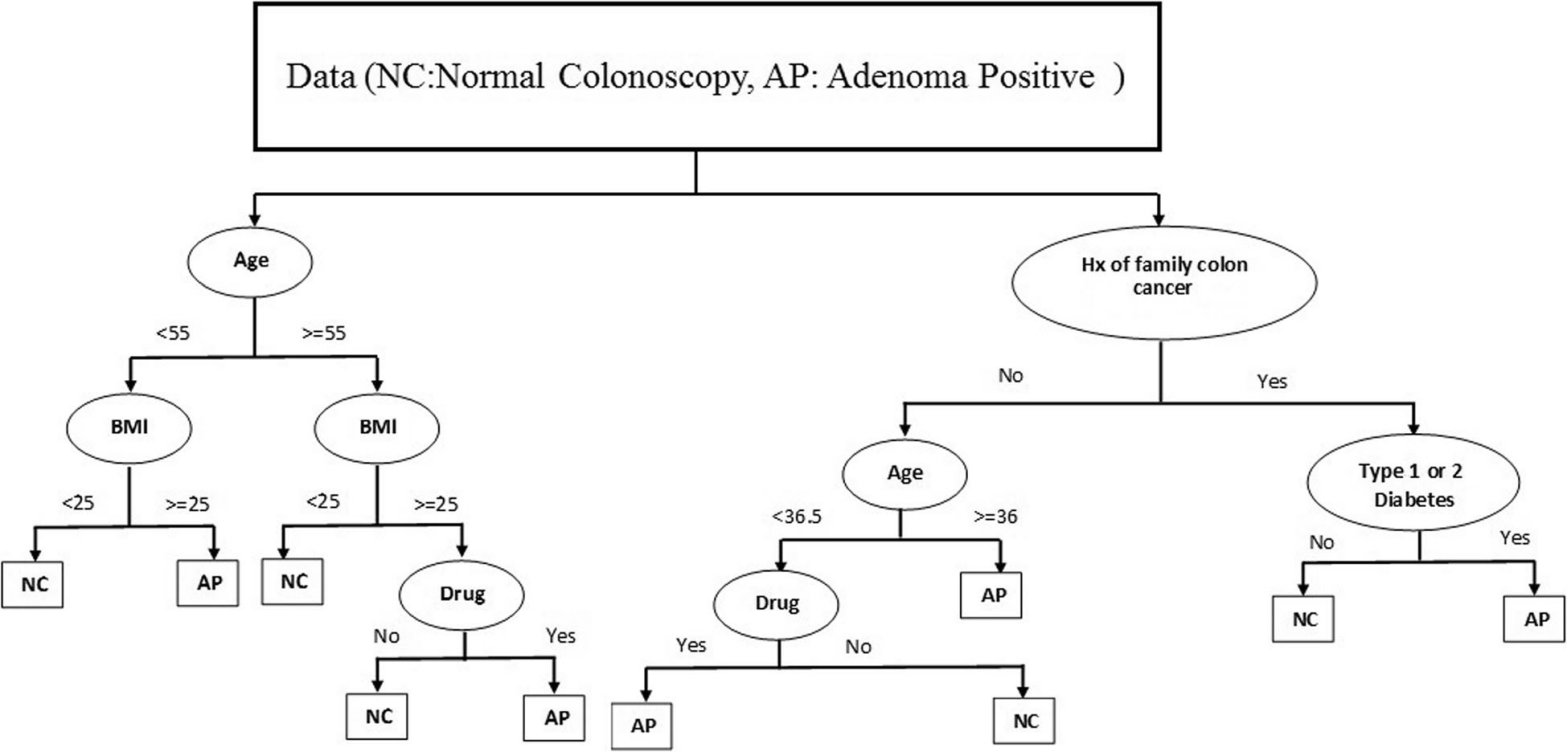
The result of decision tree based on high risk factors (BMI (kg/m2) ≥25, age (11-85 yr), type 1 or 2 diabetes, family history of colon cancer and drug abuse)

In this paper, after discovering higher risk of colon adenoma, we assessed the prediction performance of five classification methods (DT, RF, NN, kNN and SVM) towards the discrimination between normal colonoscopy and adenoma positive groups. Classification methods, were used to categorize a set of observations into pre-defined classes based on a set of variables. Classification accuracy and root mean squared error were the main criterions for evaluating the classification and prediction of samples in the test phase. We evaluated the five classification methods on higher risk factors of colon adenoma and normal colonoscopy data. The performance results of five classification methods were presented in Fig. 2. The experimental results for each classification method on higher risk factors between colon adenoma and normal colonoscopy data were more than 82% and less than 0.42 for the percentage of classification accuracy and root mean squared error, respectively. In Fig. 3, the hierarchical structure generated by DT method could be used to classify individuals based on risk factors identified as BMI (kg/m2) ≥25, age (11–85 yr) as well as diabetes, family history of colon cancer and drug abuse, which are binary variables to normal or adenoma positive groups.

## Discussion

Recent studies have determined that obesity is a clear potential risk factor for a variety of malignancies. It has been previously showed that approximately 50% of patients with cancer had an abnormally high BMI [33]. In addition to genetic and environmental factors which contribute to CRC development, some studies assumed gender and ethnicity as predisposing factors for CRC [34, 35]. In the current study, the occurrence of obesity measured based on BMI in the adenoma positive patient group was significantly higher than the control group although there was no notable association between obesity and adenocarcinoma.

Obesity could be evaluated through several different solid anthropometric indexes such as BMI, waist circumference (WC) and waist-to-hip ratio (WHR). In a recent study, Wambui et al. showed that WC as compared to BMI was a better predictor for advanced colorectal neoplasia. The study demonstrated that subjects who were overweight at the age of 21 had a higher risk of CRC than individuals with a normal BMI. Thus, they concluded that maintaining an unhealthy BMI and WC might raise the risk of CRC [36]. The WC is a stronger predictor for CRC risk than BMI but this is still controversial and has not been confirmed [6].

It has been revealed that visceral fat (abdominal fat) is associated with insulin impairment and high IGF2 serum level [35]. In accordance with this, a cohort study (a 23-years follow up) by Levi et al. showed that adolescence (male or female) with overweight or obesity condition were prone to colon and rectal cancer [37]. Brenner et al. indicated that the increasing incidence of CRC in younger adults might be associated with a prominent etiological factor which is obesity [38]. Jensen et al. showed that in 257.623 children, the childhood BMI and height were significantly associated with colon cancer. In other words taller and heavier children were prone to colon cancer in compare to normal-ranged participants [39]. Hanyuda et al. found that the association between BMI and CRC risk significantly differs depending on the presence or absence of poorly-differentiated foci. In the absence of poorly-differentiated foci, high BMI was associated with higher risk of CRC [40]. In a study by Shaukat et al., increase BMI was related to long-term colorectal mortality while reduced BMI could modulate the risk of cancer mortality [41]. Dong et al. conducted a meta-analysis study in 12,837 CRC cases. They showed that abdominal obesity was associated with CRC. They found that increased WC and WHR were profoundly associated with risk of CRC [6]. The underlying mechanism leading to cancer is still under investigation. It is assumed that adipose tissue produces different types of hormones and pro-inflammatory cytokines including IL-6, TNF-α, leptin and adiponectin, which could provide desirable micro-environmental inflammatory conditions for cancerous cells [22, 25]. Besides, it was shown that high levels of IL-23 and IL-10 in serum [29, 42] and IL-8 and IL-6 in the microenvironment are associated with progression of CRC [43–45]. Recent investigations have highlighted the role of IGF in CRC. IGF1 and IGF2 have been associated with numerous GI cancers [46, 47]. Several studies have elucidated that serum level and loss of imprinting of IGF2 were associated with advanced colorectal adenoma and poor prognosis in advanced stages of CRC, respectively [48–50].

In the current study, we also demonstrated an association between colon adenoma and diabetes (type 1 or 2), suggesting that diabetes could be a risk factor for adenoma and not for CRC. In this regard, in a cohort study, diabetes mellitus was not associated with any cancer such as CRC [51, 52]. It appears that diabetes mellitus does not decrease the survival of the CRC patients and CRC does not have a significant impact on glucose level of patients with diabetes mellitus [53, 54]. In a study with 3000 CRC cases which were followed up to 32 years, type 2 diabetes was significantly associated with high risk of CRC in comparison to controls but only among men [55]. In contrast, recent studies emphasize the relationship between diabetes and CRC. He et al. conducted a perspective cohort of 199,143 participants, indicating that there was a significant risk of CRC in diabetic patients as compared to non-diabetics ones [54] and especially those lower than 65 years and non-white people [55]. Consistent with this study, Overbeek et al, in 55,000 patients with type 2 diabetes and 215,000 matched controls demonstrated that both men and women with diabetes had higher chance of developing CRC [56]. This discrepancy between studies is not well explained yet.

This report was a retrospective study, which possesses several limitations such as sampling error, lack of waist circumference and waist-to-hip measurement in order to compare with BMI. For future directions, these measurements could be recorded and patient follow up will also be informative. Besides, multi-center studies could be performed to increase power of the study, and to conduct studies of high scientific quality. Besides the healthcare/policy decision making could benefit from the results of these studies for CRC screening programs.

## Conclusion

This report showed that there were significant differences in age distribution and BMI between case and control groups. This report demonstrates a strong association between colon adenoma and positive a history of type 1 and type 2 diabetes, or familial history of colon cancer. We confirmed that both diabetes and obesity (BMI ≥25 kg/m^2^) increase the risk of precancerous lesions. Therefore, such patients may consider screening for CRC at an earlier age although controversies still exist.

## Data Availability

The datasets used and/or analysed during the current study available from the corresponding author on reasonable request.

## Abbreviations

CRC: Colorectal cancer
DOR: Diagnostic odds ratio
GI: Gastrointestinal
SD: Standard deviation
WHR: Waist-to-hip ratio

## References

[1] Bardou M, Barkun AN, Martel M (2013). Obesity and colorectal cancer. Gut.

[2] Maskarinec G (2015). Excess body weight and colorectal cancer survival: the multiethnic cohort. Cancer Causes Control.

[3] Kormi SMA, Ardehkhani S, and Kerachian MA. New insights into colorectal cancer screening and early detection tests. 2017;6(2):63–68.

[4] Bray F (2018). Global cancer statistics 2018: GLOBOCAN estimates of incidence and mortality worldwide for 36 cancers in 185 countries. CA Cancer J Clin.

[5] Davari M (2012). The direct medical costs of colorectal Cancer in Iran; analyzing the Patient's level data from a Cancer specific Hospital in Isfahan. Int J Prev Med.

[6] Dong Y (2017). Abdominal obesity and colorectal cancer risk: systematic review and meta-analysis of prospective studies. Biosci Rep.

[7] Ma Y (2013). Obesity and risk of colorectal cancer: a systematic review of prospective studies. PLoS One.

[8] Hessami Arani S, Kerachian MK. Rising rates of colorectal cancer among younger Iranians: is diet to blame? Curr Oncol. 2017;24(2):e131–e137. 10.3747/co.23.3226 PMC5407876 28490936

[9] Arnold M (2017). Global patterns and trends in colorectal cancer incidence and mortality. Gut.

[10] Daniel C (2016). Severe obesity prior to diagnosis limits survival in colorectal cancer patients evaluated at a large cancer Centre. Br J Cancer.

[11] Siegel EM (2010). The effects of obesity and obesity-related conditions on colorectal cancer prognosis. Cancer Control.

[12] Doubeni CA (2012). Contribution of behavioral risk factors and obesity to socioeconomic differences in colorectal cancer incidence. J Natl Cancer Inst.

[13] Stone RAT (2017). The association of dietary quality with colorectal cancer among normal weight, overweight and obese men and women: a prospective longitudinal study in the USA. BMJ Open.

[14] World Health Organization. Global health observatory data repository: Total NCD mortality data by country 2018-06-25.

[15] Willett WC (2005). Diet and cancer: an evolving picture. Jama.

[16] Reedy J (2008). Index-based dietary patterns and risk of colorectal cancer: the NIH-AARP diet and health study. Am J Epidemiol.

[17] Ho GY, et al. Adipokines linking obesity with colorectal cancer risk in postmenopausal women. Cancer Res. 2012;72(12):3029–37. 10.1158/0008-5472.CAN-11-2771 PMC3790260 22511581

[18] Campbell PT (2010). Case–control study of overweight, obesity, and colorectal cancer risk, overall and by tumor microsatellite instability status. J Natl Cancer Inst.

[19] Tao W (2017). Colorectal cancer prognosis following obesity surgery in a population-based cohort study. Obes Surg.

[20] Hussan H (2016). Morbid obesity is associated with increased mortality, surgical complications, and incremental health care utilization in the peri-operative period of colorectal cancer surgery. World J Surg.

[21] Martinez-Useros J, Garcia-Foncillas J (2016). Obesity and colorectal cancer: molecular features of adipose tissue. J Transl Med.

[22] Grivennikov SI, Karin M (2011). Inflammatory cytokines in cancer: tumour necrosis factor and interleukin 6 take the stage. Ann Rheum Dis.

[23] Tilg H, Moschen AR. Adipocytokines: mediators linking adipose tissue, inflammation and immunity. Nat Rev Immunol. 2006;6(10):772–83. 10.1038/nri1937 16998510

[24] Liu Z (2012). Diet-induced obesity elevates colonic TNF-α in mice and is accompanied by an activation of Wnt signaling: a mechanism for obesity-associated colorectal cancer. J Nutr Biochem.

[25] Kim SH, Reaven GM (2008). Insulin resistance and hyperinsulinemia: you can't have one without the other. Diabetes Care.

[26] Peeters PJ, et al. The risk of colorectal cancer in patients with type 2 diabetes mellitus: associations with treatment stage and obesity. Diabetes Care. 2015;38(3):495–502. 10.2337/dc14-1175 25552419

[27] Harding JL (2015). Cancer risk among people with type 1 and type 2 diabetes: disentangling true associations, detection bias, and reverse causation. Diabetes Care.

[28] Mills KT, et al. Diabetes and colorectal cancer prognosis: a meta-analysis. Dis Colon Rectum. 2013;56(11):1304–19. 10.1097/DCR.0b013e3182a479f9 PMC3800045 24105007

[29] Hamilton SR, Aaltonen LA. Pathology and genetics of tumours of the digestive system. Vol. 2. 2000: IARC press Lyon.

[30] Ashktorab H (2013). Role of life events in the presence of colon polyps among African Americans. BMC Gastroenterol.

[31] Hijazi H, Chan C. A classification framework applied to cancer gene expression profiles. J Healthcare Eng. 2013;4(2):255–83. 10.1260/2040-2295.4.2.255 PMC3873740 23778014

[32] Kourou K (2015). Machine learning applications in cancer prognosis and prediction. Comput Struct Biotechnol J.

[33] Arnold M (2018). Cancers in France in 2015 attributable to high body mass index. Cancer Epidemiol.

[34] Karunanithi S (2017). RBP4-STRA6 pathway drives cancer stem cell maintenance and mediates high-fat diet-induced colon carcinogenesis. Stem Cell Rep.

[35] Ashktorab H (2014). BMI and the risk of colorectal adenoma in African-Americans. Obesity.

[36] Gathirua-Mwangi WG (2017). Changes in adult BMI and waist circumference are associated with increased risk of advanced colorectal Neoplasia. Dig Dis Sci.

[37] Levi Z (2017). Adolescent body mass index and risk of colon and rectal cancer in a cohort of 1.79 million Israeli men and women: a population-based study. Cancer.

[38] Brenner DR (2017). Increasing colorectal cancer incidence trends among younger adults in Canada. Prev Med.

[39] Jensen BW (2017). Childhood body mass index and height in relation to site-specific risks of colorectal cancers in adult life. Eur J Epidemiol.

[40] Hanyuda A (2017). Body mass index and risk of colorectal carcinoma subtypes classified by tumor differentiation status. Eur J Epidemiol.

[41] Shaukat A (2017). BMI is a risk factor for colorectal Cancer mortality. Dig Dis Sci.

[42] Stanilov N, et al. Advanced colorectal cancer is associated with enhanced IL-23 and IL-10 serum levels. Lab Med. 2010;41(3):159–63.

[43] Lee Y, et al. Interleukin-8 and its receptor CXCR2 in the tumour microenvironment promote colon cancer growth, progression and metastasis. Br J Cancer. 2012;106(11):1833–41. 10.1038/bjc.2012.177 PMC3364111 22617157

[44] Ning Y (2011). Interleukin-8 is associated with proliferation, migration, angiogenesis and chemosensitivity in vitro and in vivo in colon cancer cell line models. Int J Cancer.

[45] Shiga K (2016). Preoperative serum interleukin-6 is a potential prognostic factor for colorectal cancer, including stage II patients. Gastroenterol Res Pract.

[46] Hoyo C (2012). IGF2R genetic variants, circulating IGF2 concentrations and colon cancer risk in African Americans and whites. Dis Markers.

[47] Doyle SL, et al. IGF-1 and its receptor in esophageal cancer: association with adenocarcinoma and visceral obesity. Am J Gastroenterol. 2012;107(2):196–204. 10.1038/ajg.2011.417 22146489

[48] Gao Y (2012). Serum IGF1, IGF2 and IGFBP3 and risk of advanced colorectal adenoma. Int J Cancer.

[49] Liou J-M (2010). Plasma insulin-like growth factor-binding protein-2 levels as diagnostic and prognostic biomarker of colorectal cancer. J Clin Endocrinol Metabol.

[50] Unger C, et al. Stromal-derived IGF2 promotes colon cancer progression via paracrine and autocrine mechanisms. Oncogene. 2017;36(38):5341–55. 10.1038/onc.2017.116 28534511

[51] Chubak J (2018). Risk of colon cancer recurrence in relation to diabetes. Cancer Causes Control.

[52] Morikawa T (2011). Association of CTNNB1 (β-catenin) alterations, body mass index, and physical activity with survival in patients with colorectal cancer. Jama.

[53] Karlin NJ (2018). Survival and glycemic control in patients with colorectal cancer and diabetes mellitus. Future Sci.

[54] He J (2010). The association of diabetes with colorectal cancer risk: the multiethnic cohort. Br J Cancer.

[55] Restifo D, et al. Differential relationship between colorectal Cancer and diabetes in a nationally representative sample of adults. J Diabetes Complicat. 2018;32(9):819–23. 10.1016/j.jdiacomp.2018.06.007 PMC8011301 30099983

[56] Overbeek JA, et al. Sex-and site-specific differences in colorectal cancer risk among people with type 2 diabetes. Int J Colorectal Dis. 2019;34(2):269–76. 10.1007/s00384-018-3191-7 PMC6331739 30421309

